# Crystal structure of human cytosolic aspartyl-tRNA synthetase, a component of multi-tRNA synthetase complex

**DOI:** 10.1002/prot.24306

**Published:** 2013-04-23

**Authors:** Kyung Rok Kim, Sang Ho Park, Hyoun Sook Kim, Kyung Hee Rhee, Byung-Gyu Kim, Dae Gyu Kim, Mi Seul Park, Hyun-Jung Kim, Sunghoon Kim, Byung Woo Han

**Affiliations:** 1 Research Institute of Pharmaceutical Sciences Department of Pharmacy College of Pharmacy, Seoul National UniversitySeoul, 151-742, Korea; 2 Medicinal Bioconvergence Research Center, Seoul National UniversitySeoul, 151-742, Korea; 3 Department of Pharmacy College of Pharmacy, Chung-Ang UniversitySeoul, 156-756, Korea

**Keywords:** aspartyl-tRNA synthetase, multi-tRNA synthetase complex, N-helix, crystal structure

## Abstract

Human cytosolic aspartyl-tRNA synthetase (DRS) catalyzes the attachment of the amino acid aspartic acid to its cognate tRNA and it is a component of the multi-tRNA synthetase complex (MSC) which has been known to be involved in unexpected signaling pathways. Here, we report the crystal structure of DRS at a resolution of 2.25 Å. DRS is a homodimer with a dimer interface of 3750.5 Å^2^ which comprises 16.6% of the monomeric surface area. Our structure reveals the C-terminal end of the N-helix which is considered as a unique addition in DRS, and its conformation further supports the switching model of the N-helix for the transfer of tRNA^Asp^ to elongation factor 1α. From our analyses of the crystal structure and post-translational modification of DRS, we suggest that the phosphorylation of Ser146 provokes the separation of DRS from the MSC and provides the binding site for an interaction partner with unforeseen functions.

## INTRODUCTION

Aminoacyl-tRNA synthetases (AARSs) catalyze the attachment of respective amino acid substrate to its cognate tRNA through a two-step reaction.^1^ An intermediate adenylate is formed from amino acid and ATP in the first step, and the amino acid is charged to the ribose of the terminal adenine of tRNA in the second step. Although AARSs catalyze the same type of reaction, they differ in amino acid sequence, size, three-dimensional structure, and oligomeric state. AARSs can be classified into two major classes; Classes I and II synthetase.^2^ The Class I synthetase contains the representative Rossmann fold that binds ATP and the Class II synthetase adopts a core antiparallel β-sheet surrounded by α-helices and three unique conserved motifs namely Motifs 1, 2, and 3.^2^

For protein synthesis, AARSs play a basic cellular role not only in cytosol but also in mitochondria and most AARSs function distinctively in either location, and thus there are cytosolic or mitochondrial AARSs. In human, there are cytosolic and mitochondrial aspartyl-tRNA synthetases (DRS and DRS2), which share only 22.9% sequence identity. It has been known that DRS2 is associated with leukoencephalopathy with brain stem and spinal cord involvement and high lactate (LBSL)^3^ and its structure revealed the function of the additional motif in the catalytic domain.^4^ In the case of DRS, it is one of the components that forms the multi-tRNA synthetase complex (MSC) in higher eukaryotes.^5^

The MSC has been regarded as a reservoir for almost half of the cytosolic tRNA synthetases and it has been known to switch the canonical translational function and additional functions which are often observed in higher eukaryotes.^6^,^7^ In the MSC, DRS is known to interact with the AARS-interacting multifunctional protein 2 (aminoacyl-tRNA synthetase-interacting multifunctional protein AIMP2/p38)^8^,^9^ and the lysyl-tRNA synthetase (KRS).^10^ The N-terminal extension of DRS, KRS, and asparaginyl-tRNA synthetase (NRS) is unique in the Class II synthetases and further classifies them into the subclass IIb.^11^ The nuclear magnetic resonance (NMR) structure of the 21-residue N-terminal extension in DRS revealed that the N-terminal flexible β-turn followed by the amphipathic C-terminal helix induces the nonspecific tRNA binding and gives a force to transfer its charged tRNA to elongation factor 1α (EF-1α).^12^–^14^

In this study, we present the crystal structure of human cytosolic DRS at 2.25 Å. We show that DRS forms a homodimer with the N-terminal extension, anticodon-binding domain, hinge region, and catalytic domain. Analyses of our crystal structure and post-translational modification (PTM) shed lights on the molecular basis of the association and dissociation of DRS with the MSC.

## MATERIALS AND METHODS

### Cloning, protein expression, and purification

Human cytosolic full-length DRS (501 amino acids) was cloned into the pET-28a(+) vector containing the N-terminal His_6_-tag (Novagen). The recombinant protein was transformed into *Escherichia coli* Rosetta2(DE3)pLysS strain. DRS was induced by 0.5 m*M* isopropyl 1-thio-β-D-galactopyranoside and incubated for 6 h at 310 K using Luria Broth culture medium. The harvested cell was sonicated with lysis buffer containing 20 m*M* of Tris-HCl (pH 7.5), 500 m*M* of NaCl, 35 m*M* of imidazole, and 1 m*M* of phenylmethanesulfonyl fluoride. The lysates were centrifuged at 35,000*g* for 50 min to remove the cell debris and denatured proteins. The supernatant was loaded onto a HiTrap Chelating HP column (GE Healthcare) and eluted with linear gradient 50–500 m*M* of imidazole following equilibration with 50 m*M* of imidazole. The protein was diluted with a buffer containing 50 m*M* of 4-(2-hydroxyethyl)-1-piperazineethanesulfonic acid (HEPES)–NaOH (pH 7.0), 50 m*M* of NaCl, 1 m*M* of dithiothreitol, and 5% of glycerol, and further purified using the ion exchange chromatography with a HiTrap Q HP column (GE Healthcare). The final purification step was the size-exclusion chromatography with a HiLoad 16/600 Superdex 200 prep grade column (GE Healthcare) equilibrated with 50 m*M* of HEPES–NaOH (pH 7.0), 200 m*M* of NaCl, 5% of glycerol, and 1 m*M* of dithiothreitol. For crystallization, the purified protein was concentrated to 11.1 mg mL^−1^.

### Crystallization, data collection, and structure determination

DRS crystals were grown by the sitting-drop vapor-diffusion method at 295 K by mixing equal volumes of the purified protein and the reservoir solution containing 8% v/v tacsimate (pH 8.0), and 20% w/v polyethylene glycol 3350. For diffraction data collection, crystals were soaked in the cryoprotectant solution containing 20% v/v glycerol added to the reservoir solution. X-ray diffraction data of the crystal were collected at the synchrotron BL-5A at the Photon Factory, Japan. The structure was solved by molecular replacement method with the structure of *Saccharomyces cerevisiae* DRS containing the anticodon-binding domain, hinge region, and catalytic domain (PDB ID: 1ASZ)^15^ as a phasing model using *MOLREP*.^16^ The structure was completed using alternate cycles of manual building in *WinCoot*^17^ and refinement in *REFMAC*.^18^ All refinement steps were monitored using an *R*_free_ value based on 5.0% of the independent reflections. The stereochemical quality of the final model was assessed using *MolProbity*.^19^ The data collection and refinement statistics are summarized in Table I.

**Table I:** Statistics for Data Collection, Phasing, and Model Refinement

Data collection^a^	Human cytosolic DRS
Space group	*P*2_1_
*Cell dimensions*	
*a*, *b*, *c* (Å)	54.89, 141.92, 68.50
*α*, *β*, *γ* (°)	90, 102.19, 90
*Data set*	
X-ray wavelength (Å)	1.0000
Resolution (Å)^b^	50.00–2.25 (2.29–2.25)
Total/unique reflections	177,246/48,428
Completeness (%)	99.3 (95.4)
*R*_merge_ (%)^c^	11.0 (50.2)
*Refinement*	
Resolution (Å)	50.00–2.24
*R*_work_^d^/*R*_free_^e^ (%)	19.7/22.8
*No. of nonhydrogen atoms/mean B-factor (Å^2^)*	
Protein	6968/33.7
Water	354/40.2
Glycerol	48/52.5
Poor rotamers (%)^f^	3.1
*Ramachandran plot analysis (%)*	
Most favored regions	96.9
Additional allowed regions	3.1
Disallowed regions	0
*R.m.s.d. from ideal geometry*	
Bond lengths (Å)	0.010
Bond angles (°)	1.290

a Data collected at the synchrotron BL-5A at the Photon Factory, Japan.

b Numbers in parentheses indicate the highest resolution shell.

c *R*_merge_ = Σ_*h*_ Σ_*i*_ |*I*(*h*)_*i*_ − <*I*(*h*)>|/Σ_*h*_ Σ_*i*_ *I*(*h*)_*i*_, where *I*(*h*) is the observed intensity of reflection *h*, and <*I*(*h*)> is the average intensity obtained from multiple measurements.

d *R*_work_ = Σ ||*F*_o_| − |*F*_c_| |/Σ |*F*_o_|, where |*F*_o_| is the observed structure factor amplitude and |*F*_c_| is the calculated structure factor amplitude.

e *R*_free_ = *R*-factor based on 5.0% of the data excluded from refinement.

f Values obtained using *MolProbity*.

### PTM analysis

Purified N-terminal OneSTrEP-tagged DRS and coeluted interaction partners of that, overexpressed and purified from HEK293T cells, were digested with sequencing grade gold-trypsin (Promega) after 1D-SDS PAGE/Coomassie blue staining. Tryptic peptides were analyzed with the LTQ Velos Orbitrap mass spectrometer equipped with an electron transfer dissociation source after an online reversed-phase chromatography. To improve sequencing coverage, we applied a data-dependent decision tree to select for collision-induced dissociation or electron-transfer dissociation fragmentation depending on the charged states, respectively. Protein identification was accomplished using the Sorcerer™-SEQUEST® (Sage-N Research), and searches were performed against the IPI human DB v3.87 fasta. The carbamidomethylation (+57.021 Da) of Cys is set as a static modification, and the following variable modification were allowed: GlyGly/+114.043 Da (Lys), Acetyl/+42.011 Da (Lys), HexNAc/+203.079 Da (Asn, Ser, Thr), Phospho/+79.966 Da (Ser, Thr, Tyr), Oxidation/+15.995 Da (Met), deamidated/+0.984 Da (Asn, Gln).

### Data deposition

The coordinate and structure factors for human cytosolic DRS have been deposited in the RCSB Protein Data Bank, accession code 4J15.

## RESULTS AND DISCUSSION

### Overall structure and oligomeric state of DRS

**Figure 1:**
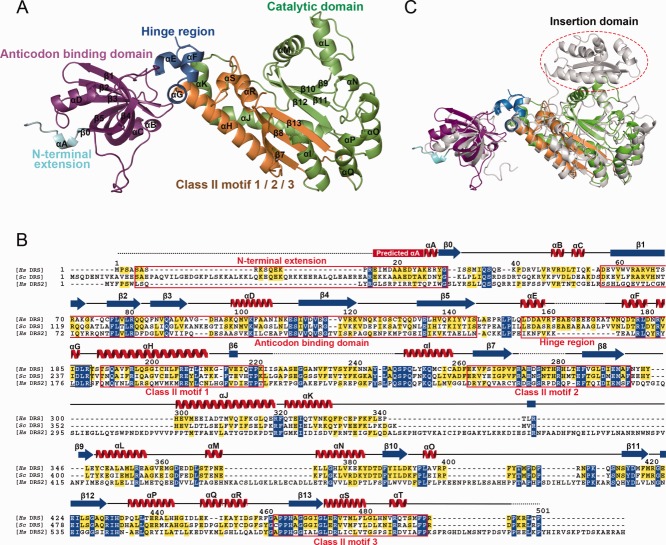
Overall structure of DRS. (**A**) DRS monomer. The N-terminal extension, anticodon-binding domain, hinge region, catalytic domain, and motifs are colored and labeled in cyan, magentas, blue, green, and orange, respectively. (**B**) Sequence alignment of human cytosolic DRS (*Hs* DRS) with *S. cerevisiae* DRS (*Sc* DRS) and human mitochondrial DRS (*Hs* DRS2). Strictly conserved residues are highlighted with blue-shaded boxes, and moderately conserved residues are shown as yellow-shaded boxes. The secondary structure of human DRS is shown on top of the sequence alignment. α-Helix, β-sheet, connecting region, and disordered region are represented by red spiral, blue arrow, black line, and dotted line, respectively. (**C**) Structural comparison of human cytosolic and mitochondrial DRSs. Human cytosolic DRS is shown as in (A) and mitochondrial DRS is colored in gray. The red dotted oval indicates the insertion domain of mitochondrial DRS.

The crystal structure of human cytosolic DRS was determined at a resolution of 2.25 Å by molecule replacement method with the structure of *S. cerevisiae* DRS containing the anticodon-binding domain, hinge region, and catalytic domain (PDB ID: 1ASZ)^15^ as a phasing model. DRS contains a homodimer in the asymmetric unit and the dimer interface area is 3750.5 Å^2^ which comprises 16.6% of the monomeric surface area calculated from Protein Interfaces, Surfaces, and Assemblies service.^20^ Our crystal structure includes all the Class II AARS domains: anticodon-binding domain, hinge region, and catalytic domain. In addition, the N-terminal extension, which is a distinct domain in mammalian DRS, could be partially modeled including the C-terminal three residues of the characteristic helix motif [Fig. 1(A)]. Structural analyses on this N-terminal extension will be further discussed with the previous NMR structure below.

The anticodon-binding domain of DRS (residues, 57–146) adopts the oligonucleotide binding-fold (OB-fold) that is composed of a five-stranded antiparallel β-sheet connected by helices and loops (β1–β5) to form a closed β-barrel. The catalytic domain (residues, 189–497) contains 13 α-helices (αH–αT) and 8 β-strands (β6–β13) which constitute all the three conventional Class II AARS motifs: Motifs 1, 2, and 3.^2^ The hinge region (residues, 156–188) plays an essential role in the connection of the anticodon-binding domain and the catalytic domain. In the middle of the hinge region, residues 163–172 could not be modeled owing to the lack of the electron density and the disordered residues are considered as a part of binding region to the ribose-phosphate backbone in the D-stem of tRNA^Asp^, compared with the known *S. cerevisiae* DRS–tRNA^Asp^ complex structure.^15^ In addition, residues 224–247 in the flipping loop and residues 273–282 in the Motif 2 could not be observed in our crystal structure [Fig. 1(B)]. These regions are known to be dynamic without its cognate tRNA and recognize its tRNA in an induced-fit manner.^21^

When the anticodon-binding domain, hinge region, and catalytic domain in our structure were independently superimposed with those of the *S. cerevisiae* DRS–tRNA^Asp^ complex structure, then the three domains were structurally well conserved with the root mean square deviation (r.m.s.d.) values of 1.08, 1.80, and 0.97 Å, respectively. In the anticodon-binding domain, three β-strands (β1, β2, and β3) could bind to the anticodon loop of tRNA^Asp^ which is composed of GUC elements. In the hinge region, short helices containing Asp158 and Asn175 could interact with the D-stem (U11, U12) of tRNA^Asp^. In the catalytic domain, the flipping loop and the Class II Motif 2 play a key role in the interaction of DRS with the 3′-end of tRNAAsp. Detailed representations of the superposition results are shown in Supporting Information Figure S1.

Recently, the crystal structure of human mitochondrial DRS (DRS2) was solved at a resolution of 3.7 Å (PDB ID: 4AH6).^4^ Human DRS and DRS2 share only 22.9% sequence identity [Fig. 1(B)]. However, when our crystal structure of DRS was superposed with that of DRS2, two structures are structurally similar to each other with the r.m.s.d. distance of 1.7 Å. The anticodon-binding domain, hinge region, and catalytic domain of DRS and DRS2 are structurally conserved, with the exception of an additional motif in the catalytic domain of DRS2 which is known as the insertion domain [Fig. 1(C)]. The insertion domain of DRS2 forms the enlarged catalytic groove with more electropositive surface potential, which enables an alternate interaction network at the subunit interface between tRNA and DRS2.^4^ Interestingly, DRS2 showed a higher sensitivity than DRS for inhibitors with a nonhydrolysable adenylate moiety and its correlation with structural features has not been well understood.^22^ Structural analyses of DRS and DRS2 in complex with same adenylate analogs would elucidate a subtle role of the domain difference with respect to substrate specificity and evolutionary advantages.

### Flexible N-terminal extension of DRS

**Figure 2:**
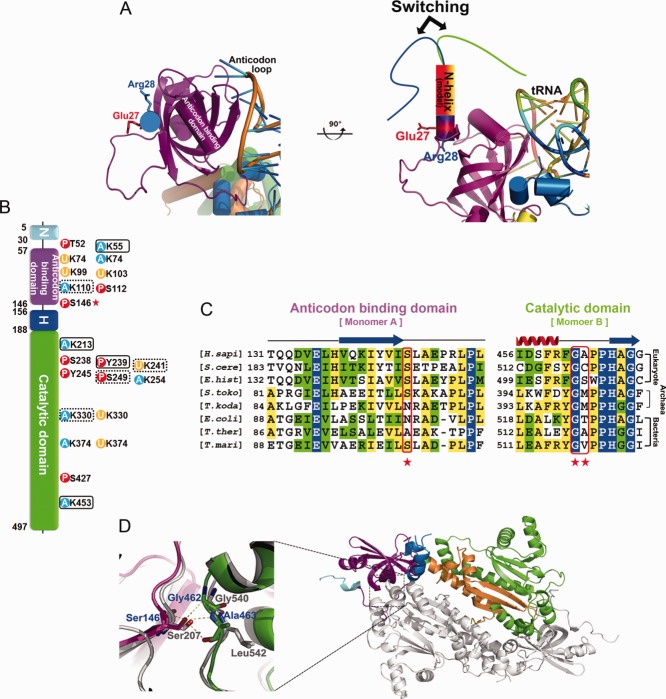
N-terminal extension, PTM, and key intermolecular interaction of DRS. (**A**) The switching model of the N-helix with our DRS structure. (**B**) PTM analyses. Acetylation, phosphorylation, and ubiquitination sites are shown as blue, red, and yellow circles, respectively. PTM sites uniquely observed in this study and residues observed both in the database and in our study are surrounded by black boxes and dotted boxes, respectively. Ser146, which is expected to play a key role in the organization of DRS, is marked with a red asterisk. (**C**) Sequence alignment of the interface residues of anticodon-binding domain and catalytic domain of DRSs from various organisms. Ser146, Gly462, and Ala463 of human DRS are marked with red asterisks. (**D**) Close-up view of Ser146 and the intermolecular interaction of DRS dimer. The structure of human DRS is superimposed with that of human KRS shown in a gray cartoon model.

In higher eukaryotes, additional domains or motifs in a specific AARS result in new functions. In the case of DRS, KRS, and NRS, they contain the N-helix that is named after the helical conformation in part of their N-terminal extension region.^11^,^23^ The previously determined NMR structure of the N-terminal extension of DRS revealed the conformational flexibility caused by the β-turn followed by one α-helix and the N-terminal extension plays a crucial role in the interaction between tRNA^Asp^ and EF-1α.^12^–^14^ In our crystal structure, the C-terminal end of the α-helix in the N-terminal extension was observed, comprising Lys26, Glu27, and Arg28 although the N-terminal region was less-ordered. To get a glimpse of the whole N-helix structure, we superposed the structurally well-resolved C-terminal end of the N-terminal extension residue Glu27 and Arg28 with the α-helix of the NMR structure, considering the helical wheel conformation (Supporting Information Fig. S2). The α-helix in the N-terminal extension is amphipathic and the hydrophilic face of the amphipathic helix could interact with positively charged residues Arg8 and Lys9 in the N-terminus by the conformational change on the flexible β-turn.^14^ Our crystal structure further supports the structural switching model of the N-terminal extension of DRS in the aid of the direct transfer of tRNA^Asp^ to EF-1α [Fig. 2(A)].

### PTM of DRS and its implication on the MSC assembly

To speculate the organization of DRS as a main interacting component with AIMP2/p38 in the MSC assembly, we searched all the known PTM data and independently implemented the PTM analyses of DRS. Our liquid chromatography tandem mass spectrometry (MS) analysis revealed two phosphorylation sites (Tyr239 and Ser249) and six acetylation sites (Lys55, 110, 213, 241, 330, and 453), respectively. Among them, the phosphorylation of Tyr239 and the acetylation of Lys55, 213, and 453 were first identified. Our findings and other PTM site information of DRS from PhosphoSite Plus database (http://www.phosphosite.org) indicated that the residues 238–254 regions of the Class II AARS are dynamically regulated by various types of modifications such as phosphorylation, acetylation, and ubiquitination [Fig. 2(B)]. For instance, although Lys241 was identified with its acetylation modification in our analysis, collected six independent MS analysis data of ubiquitin branch motif (K-e-GG) immunoaffinity beads purification studies showed that Lys241 is also modified with ubiquitin. Thus, it seems that the phosphorylation status of Ser238, Tyr239, and/or Ser249 could affect the catalytic activity, stability, or partner-binding affinity of DRS through a competitive modification event between acetylation and ubiquitination of Lys241 and/or Lys254 though it should be clarified with further studies. PTM sites mapped on the surface representation of human DRS dimer modeled with tRNA^Asp^ are shown in Supporting Information Figure S3.

In the MSC, AIMP2/p38 has been known to interact with two subcomplexes of the MSC (I: MRS, AIMP3/p18, EPRS, IRS, LRS; II: AIMP1/p43, QRS, RRS), KRS, and DRS, and the N-terminal domain of AIMP2/p38 interacts with the subcomplex II, KRS, and DRS.^9^ The N-terminal Motifs 1 and 2 of AIMP2/p38 have been recently shown to interact with the bottom groove and the symmetric groove on the KRS dimer, respectively.^24^ Based on the situational and structural similarity between KRS and DRS, we anticipated that two dimers of DRS would interact with AIMP2/p38 in the similar way of KRS regarding the association and dissociation from the MSC. In the case of KRS, Ser207 establishes the major intermolecular interaction of the KRS dimer through three hydrogen bonds between the hydroxyl group of Ser207 and the backbone of Gly540 and Leu541. Interestingly, a conformational change triggered by the phosphorylation of Ser207 switches the function of KRS from translation to transcription, provoking a new conformer and releasing KRS from the MSC.^24^ To our surprise, DRS also contains the equivalent Ser146, Gly462, and Ala463, which are highly conserved in the amino acid sequence and three-dimensional structure in higher eukaryotes [Fig. 2(C–D)]. In addition, the PTM information of DRS from the PhosphoSite Plus database shows that the phosphorylation of Ser146 was already observed with the phosphoproteome analyses of the human cell cycle using the MS.^25^ Thus, we suggest that the phosphorylation of Ser146 could initiate a conformation change of the DRS dimer and trigger an unpredicted function of DRS by releasing it from the MSC. Our structural study and PTM analyses extend the knowledge about the interaction of components in the MSC and provide fundamental information for human physiological signaling pathways related to the MSC.

## Abbreviations

AARS: aminoacyl-tRNA synthetase
AIMP: aminoacyl-tRNA synthetase-interacting multifunctional protein
DRS: aspartyl-tRNA synthetase
DRS2: mitochondrial aspartyl-tRNA synthetase
EF-1α: elongation factor 1α
KRS: lysyl-tRNA synthetase
MSC: multi-tRNA synthetase complex
NMR: nuclear magnetic resonance
NRS: asparaginyl-tRNA synthetase
PTM: post-translational modification.
