# Supplementary material for: Crystal structure of human cytosolic aspartyl-tRNA synthetase, a component of multi-tRNA synthetase complex
Source: Proteins. 2013 Apr 23;81(10):1840–6. doi: 10.1002/prot.24306 (PMC3824080; doi:10.1002/prot.24306)
Supplement: Supplementary file 1 [file prot0081-1840-SD1.docx]

Supplementary Information

**Crystal structure of human cytosolic aspartyl-tRNA synthetase, a component of multi-tRNA synthetase complex**

Running title: Crystal structure of human cytosolic DRS

Kyung Rok Kim,^1*^ Sang Ho Park,^1*^ Hyoun Sook Kim,^1^ Kyung Hee Rhee,^1^ Byung-Gyu Kim,^2^ Dae Gyu Kim,^2^ Mi Seul Park,^1^ Hyun-Jung Kim,^3^ Sunghoon Kim,^2^ and Byung Woo Han,^1†^

^1^Research Institute of Pharmaceutical Sciences, College of Pharmacy, Seoul National University, Seoul, 151-742, Korea

^2^Medicinal Bioconvergence Research Center, Seoul National University, Seoul, 151-742, Korea

^3^College of Pharmacy, Chung-Ang University, Seoul, 156-756, Korea

^*^Kyung Rok Kim and Sang Ho Park contributed equally to this work.

^†^Correspondence to: Byung Woo Han, College of Pharmacy, Seoul National University, Seoul, 151-742, Korea. E-mail: [bwhan@snu.ac.kr](mailto:bwhan@snu.ac.kr)


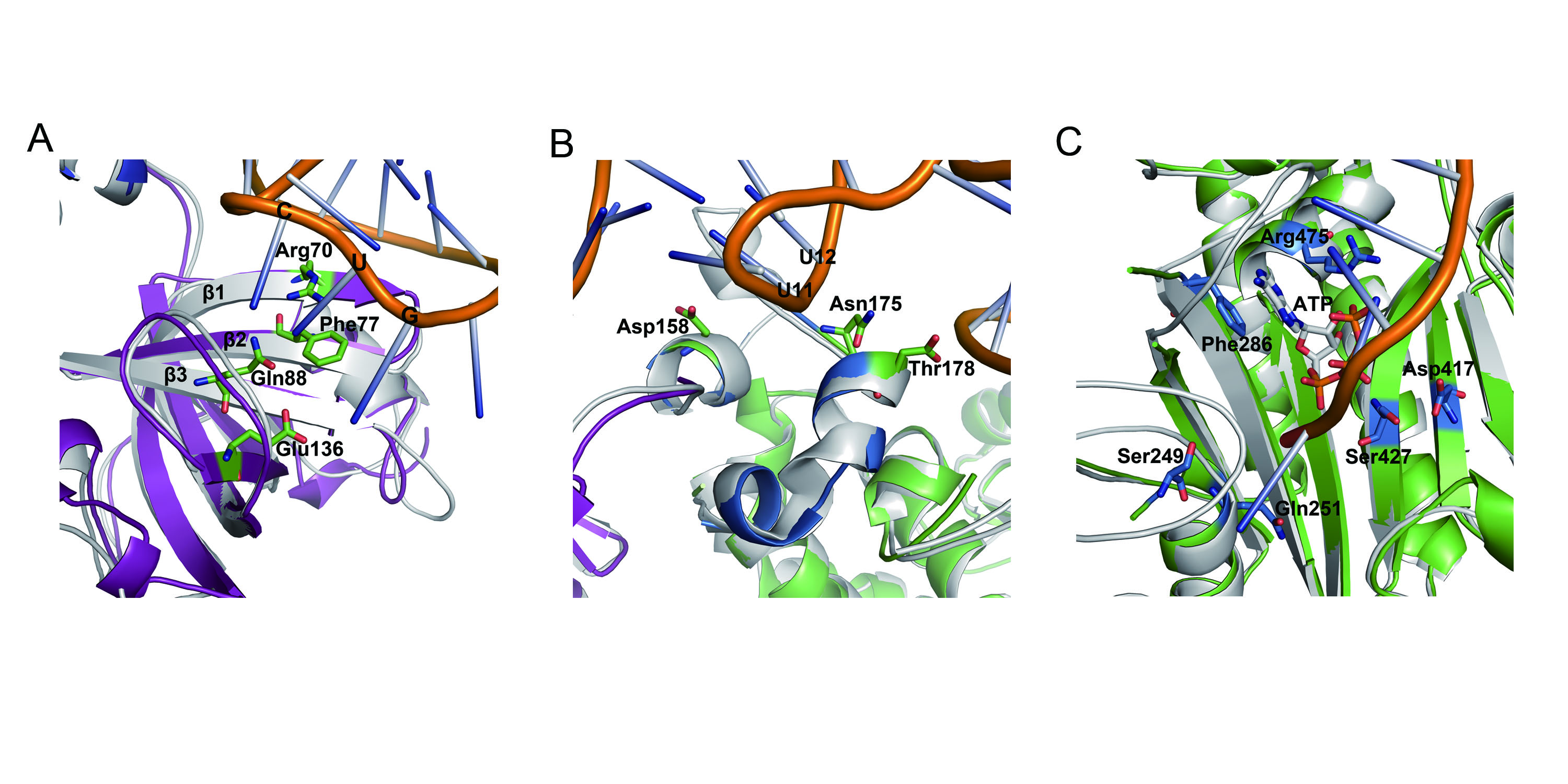


**Figure S1. Superposition of human DRS with *S. cerevisiae* DRS-tRNA^Asp^ complex.** The anticodon binding domain (**A**) and the hinge region (**B**) of human DRS are colored as in Figure 1(A) and the catalytic domain (**C**) of human DRS is represented in green. *S. cerevisiae* DRS is colored in gray and tRNA^Asp^ is shown in orange. Carbon, oxygen, nitrogen and phosphorus atoms of ATP are shown with stick model in gray, red, blue, and orange, respectively. The predicted interaction residues of human DRS with tRNA^Asp^ are labeled and shown in stick model.


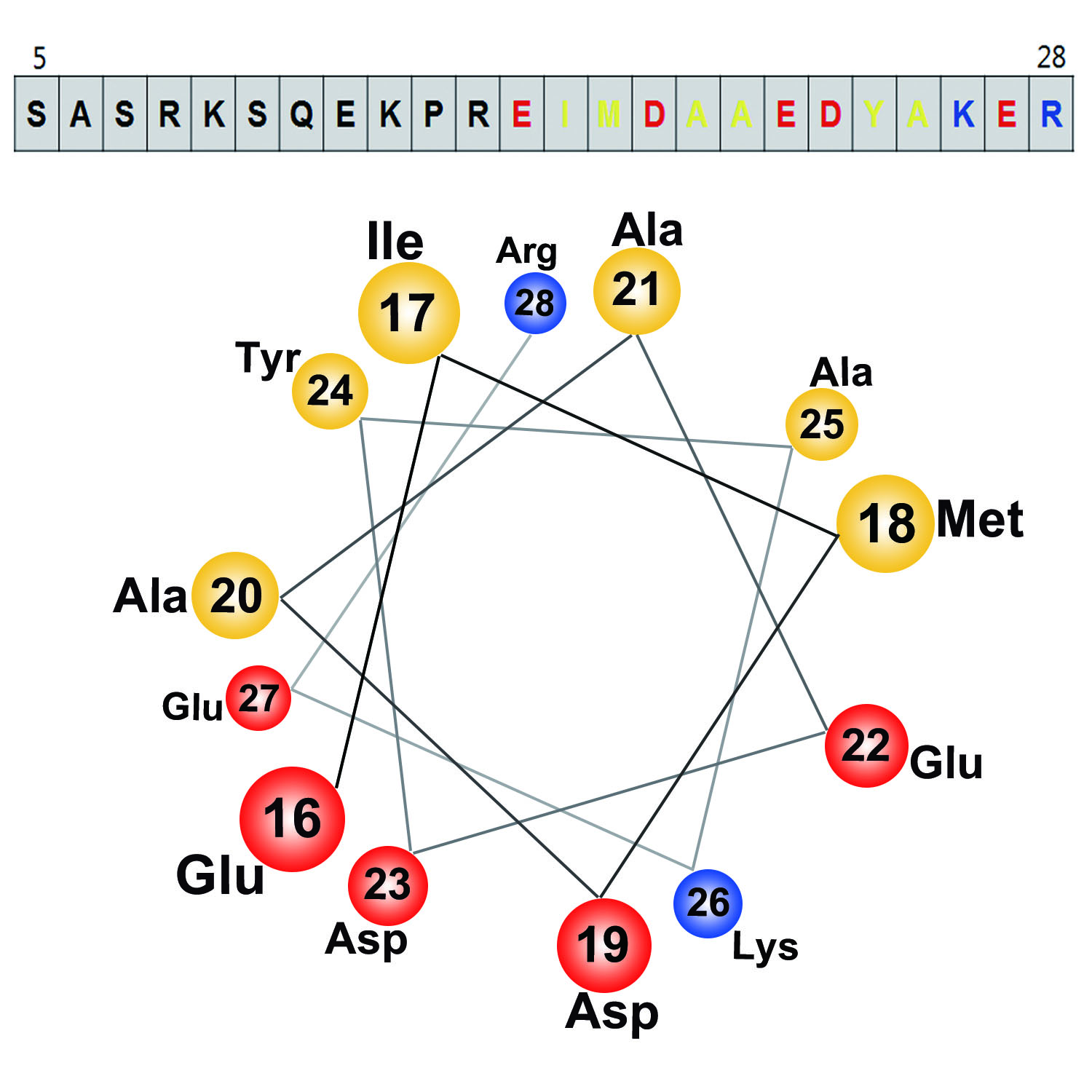


**Figure S2. Helical wheel representation of the α-helix in the N-terminal extension.** The hydrophobic, negative-charged, and positive-charged residues are colored in yellow, red, and blue, respectively.


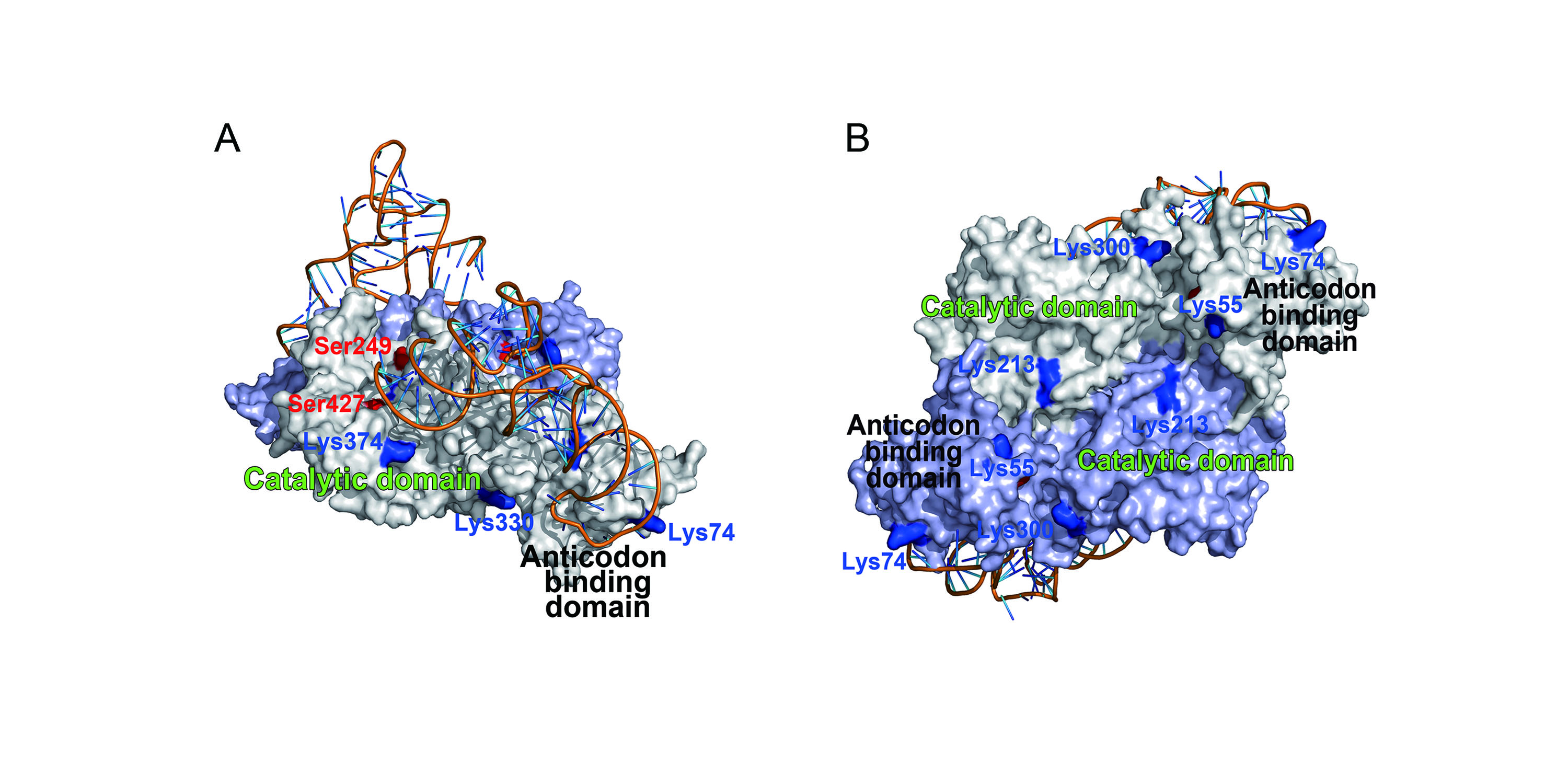


**Figure S3. PTM sites mapped on the surface representation of human DRS dimer modeled with tRNA^Asp^.** The crystal structure of human DRS dimer was superposed with that of *S. cerevisiae* DRS dimer in complex with tRNA^Asp^ and human DRS is shown as surface representation. (**A**) tRNA binding face of DRS dimer. (**B**) Bottom and symmetric groove of the DRS dimer. Acetylation and phosphorylation sites are colored in blue and red, respectively.
